# ViromeScan: a new tool for metagenomic viral community profiling

**DOI:** 10.1186/s12864-016-2446-3

**Published:** 2016-03-01

**Authors:** Simone Rampelli, Matteo Soverini, Silvia Turroni, Sara Quercia, Elena Biagi, Patrizia Brigidi, Marco Candela

**Affiliations:** Department of Pharmacy and Biotechnology, University of Bologna, Via Belmeloro 6, Bologna, 40126 Italy

**Keywords:** Virome, Microbiome, Metagenomics

## Abstract

**Background:**

Bioinformatics tools available for metagenomic sequencing analysis are principally devoted to the identification of microorganisms populating an ecological niche, but they usually do not consider viruses. Only some software have been designed to profile the viral sequences, however they are not efficient in the characterization of viruses in the context of complex communities, like the intestinal microbiota, containing bacteria, archeabacteria, eukaryotic microorganisms and viruses. In any case, a comprehensive description of the host-microbiota interactions can not ignore the profile of eukaryotic viruses within the virome, as viruses are definitely critical for the regulation of the host immunophenotype.

**Results:**

ViromeScan is an innovative metagenomic analysis tool that characterizes the taxonomy of the virome directly from raw data of next-generation sequencing. The tool uses hierarchical databases for eukaryotic viruses to unambiguously assign reads to viral species more accurately and >1000 fold faster than other existing approaches. We validated ViromeScan on synthetic microbial communities and applied it on metagenomic samples of the Human Microbiome Project, providing a sensitive eukaryotic virome profiling of different human body sites.

**Conclusions:**

ViromeScan allows the user to explore and taxonomically characterize the virome from metagenomic reads, efficiently denoising samples from reads of other microorganisms. This implies that users can fully characterize the microbiome, including bacteria and viruses, by shotgun metagenomic sequencing followed by different bioinformatic pipelines.

**Electronic supplementary material:**

The online version of this article (doi:10.1186/s12864-016-2446-3) contains supplementary material, which is available to authorized users.

## Background

Viruses constantly inhabit our body [1]. Even asymptomatic hosts harbor viral communities and interactions between viruses and the host do not always end with the death of the virus-infected cells [2, 3]. This emerging vision raises the need to explore the virome as a significant part of our biology, which can profoundly influence the host in several ways, also other than the classical viral infections [1, 2]. For example, like bacteria and fungi, certain viruses can stimulate a low level of immune responses, which are important for the long-term modulation of the human immunological but also metabolic homeostasis. In this regard, Foxman and Iwasaki [4] showed that a constant reinfection by common low-virulence viruses stimulates antiviral components of the immune system, which correlate with susceptibility to diseases, such as type 1 diabetes and asthma. On the other hand, it has been reported that the viruses usually present in the acute infection of the nasopharynx, are also commonly detected in healthy individuals [5]. Taken together, these data contribute to describe the host-virome interplay as a complex relationship that generally encompasses symbiosis, sometimes with a pathogenic outcome, and can profoundly impact on host health and disease. For these reasons, profiling the taxonomic and phylogenetic composition of such viral communities is pivotal not only to better understand their role in the biology of the human holobiont but also to open new possibilities in the interpretation of complex disorders, with the ultimate goal of eradicating them [1, 4, 6–13].

Even if the importance of the interplay among eukaryotic virome, microbiome and immune system is already evident, the available techniques for virome characterization usually underestimate the quantity and diversity of viruses in the samples [14]. For example, it is recognized that the methods for the viral isolation based on filtering procedures miss giant virus [15]. Viral communities are also difficult to be characterized since there is not a single gene common to all viral genomes, which prevents the application of analogous approaches to ribosomal DNA profiling for bacteria [2]. The viral taxonomic composition of a microbial community could be estimated from metagenomic shotgun sequencing and RNA-seq of the microbiota DNA/RNA, by detecting and assigning the viral reads to the appropriate viral taxa. Metagenomic samples contain indeed nucleic acids for bacteria, archeabacteria, eukaryotes, phages and eukaryotic viruses. However, currently the most advanced experimental procedures foresee to extract and isolate the encapsidated viral fraction [16–18] and only at a later stage, to characterize the metavirome by assembled or read-mapping approaches [19–22]. To sequence unprocessed samples and directly assign the obtained reads would instead allow a faster characterization of the virome in the context of the microbiome of origin and no risk to miss giant virus due to filtering procedures. We thus present ViromeScan, a new tool that accurately profiles viral communities and requires only few minutes to process thousands of metagenomics reads. ViromeScan works with shotgun reads to detect traces of DNA and/or RNA viruses, depending on the input sequences to be processed. ViromeScan has been developed to profile the eukaryotic viral community within the microbiome, in particular it estimates the relative abundance of viruses by filtering out the metagenomic reads of human and bacterial provenance, and mapping the remaining sequences on a hierarchical viral database. ViromeScan is available at the website http://sourceforge.net/projects/viromescan/.

## Implementation

### Workflow of the software

Once downloaded, ViromeScan locally processes the metagenome for searching eukaryotic viral sequences. Input files should be single-end or paired-end reads in fastq format (for paired-end reads compressed files in gzip, bzip2 and zip formats are also accepted) retrieved from shotgun sequencing or RNA-seq. Depending on the research strategy, ViromeScan gives users the option to choose from a range of in-house built reference databases, including human DNA virus database, human DNA/RNA virus database, eukaryotic DNA virus database and eukaryotic DNA/RNA virus database. The human virus databases contain only viruses that have the human being as the natural host; on the other hand, the eukaryotic virus databases also include viruses for vertebrates, invertebrates, fungi, algae and plants, while excluding bacteriophages. All databases are based on the complete viral genomes available on the NCBI website [23]. The NCBI IDs of the viral genomes used to build the different databases are reported in Additional file 1.

**Fig. 1:**
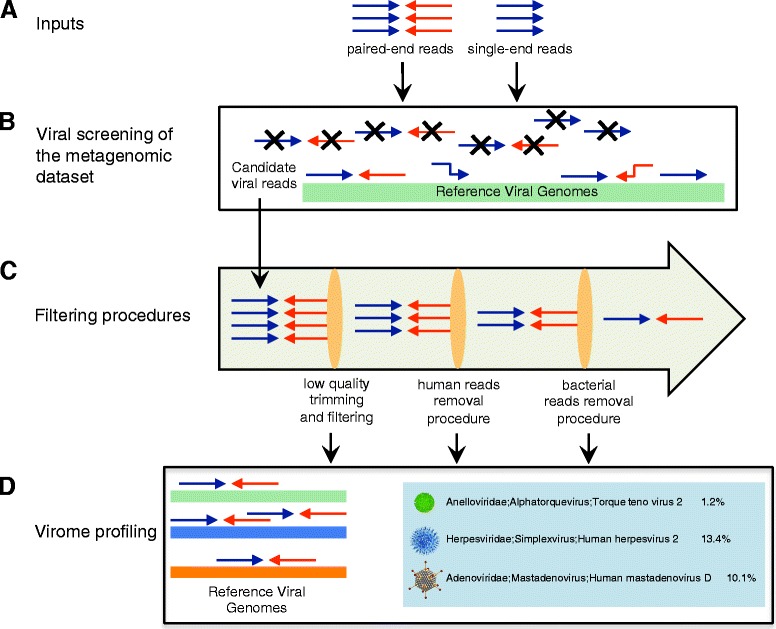
Workflow of ViromeScan. **a** Inputs are single-end reads (fastq format) or paired-end reads (fastq or compressed fastq format). **b** Candidate viral reads are identified by mapping the sequences to the selected reference database. Unmapped reads are not contained in the resulting file. **c** Three filtering procedures to trim low quality reads and completely remove human and bacterial contaminations are computed. **d** The remaining viral sequences are assigned to appropriate taxonomy and the results are tabulated as both relative abundance and read counts

The schematic description of the procedures of analysis computed by ViromeScan is provided in Fig. 1. In detail, metagenomic reads are compared to the viral genomes of the selected database using bowtie2 [24]. This first step is a complete and accurate screening of the sequences to select candidate viral reads. To perform this procedure before filtering processes allows a considerable gain of time in the subsequent parts of the pipeline, due to the reduction of the dataset to less than 1 % of the total amount of metagenomic reads. Afterwards, a quality filtering step of the candidate viral reads has been implemented as described in the processing procedure of the Human Microbiome Project (HMP) [25]. In brief, sequences are trimmed for low quality score using a modified version of the script trimBWAstyle.pl that works directly from BAM files [26]. The script is utilized to trim bases off the ends of sequences, which show a quality value of two or lower. This threshold is taken to delete all the bases with an uncertain quality as defined by Illumina’s EAMMS (End Anchored Max Scoring Segments) filter. Additionally, reads trimmed to less than 60 bp are also removed.

Since the sequences analyzed are whole-genome or RNA-seq products, it is plausible that the candidate viral reads contain a small percentage of human reads. For this reason, it is necessary to subject the sequences to the control for human contamination. As reported in the HMP procedures [27], Human Best Match Tagger (BMTagger) [28] is an efficient tool that discriminates among human, viral and microbial reads. First of all, BMTagger attempts to discriminate between human reads and the other reads by comparing the 18mers produced from the input file with those contained in the reference human database. If this process fails, an additional alignment procedure is performed to guarantee the detection of all matches with up to two errors.

Human-filtered reads may also contain an amount of bacterial sequences, which need to be filtered out to avoid biases due to bacterial contamination. Bacterial reads are identified and masked using BMTagger, the same tool utilized for the human sequence removal procedure. In particular, in order to detect bacterial sequences, human-filtered reads are screened against the genomic DNA of a representative group of bacterial taxa that are known to be common in the human body niches. See Additional file 2 for the list of bacteria included in this process. Nevertheless, the user can customize the filtering procedure by replacing the bacterial database within the ViromeScan folder with the microbial sequences of interest, associated to environments other than the human body (e.g. microbiome associated with animals, soil or water).

Finally, filtered reads are again compared to the viral genomes of the chosen hierarchical viral database using bowtie2 [24], allowing the definitive association of each virome sequence to a viral genome. For each sample analyzed, the total amount of counts is summarized in a table as number of hits and relative abundance. Additionally, graphs representing the abundances at family, genus and species level are provided, using the “graphics” and “base” R packages.

### Validation of the tool and comparison with other existing methods

Five different mock communities each containing 20 human DNA viruses at different relative abundances were built and submitted to ViromeScan for its validation. The mock communities contained also human sequences and reads of other microorganisms to test the filtering steps of the pipeline. The simulated metagenomes were composed of sequences of 100 bp randomly generated from the chosen genomic DNAs by an in-house developed script. In order to compare the performance of ViromeScan with other existing tools, the same mock samples were analyzed using Metavir [22] and blastN [29]. In particular, in the Metavir pipeline, we determined the taxonomic composition using the number of best hits normalized by genome length through the GAAS metagenomic tool [30]. The genomes used to generate the five mock communities are reported in Additional file 3.

### Case study: using ViromeScan to profile the eukaryotic DNA virome across different human body sites

Twenty metagenomic samples from HMP [27], belonging to four body sites, including stool, mid vagina, buccal mucosa and retroauricular crease, were used to illustrate the results that can be obtained by ViromeScan. The IDs of HMP samples are reported in Additional file 4. These metagenomes had been sequenced using the Illumina GAIIx platform with 101 bp paired-end reads. The entire metagenomic dataset was utilized to study the differences in the composition of the viral communities across different body sites. No ethics approval was required for any work performed in this study.

## Results and discussion

The ViromeScan software is specifically dedicated to the analysis of the virome. In particular, it can be used to detect viruses inside the microbiome from a given environment utilizing raw reads, mostly in fastq format generated by next-generation sequencing technologies. ViromeScan has the advantage of using a read-mapping approach that allows i) the characterization of the virome within a metagenome, including bacterial, eukaryotic and host sequences, without specific extraction/purification strategies, and ii) the preservation of all the information retained in the input files, information that may be lost by an assembly approach [31]. In particular, in the context of a metagenomic dataset, the viral DNA could be undersequenced due to the huge amount of bacterial and human DNA in the samples, making the assembly difficult or even impossible for viruses with a limited number of reads. However, as all the existing read-mapping approaches, ViromeScan is blind to viral sequences that are not closely related to viruses already present in the repository. For this reason and in the light of the considerable number of unknown viruses inside a metagenomic sample [32–34], the integration of a read-mapping and an assembling approach could be pursued to retrieve a more exhaustive virome profile of metagenomic samples.

ViromeScan determines the taxonomic composition of the virome by sequence alignment of the reads to completely known viral genomes, and displays the results as either number of hits or normalized hits (relative abundance). The ViromeScan classifier can be used for multiple analysis of the virome, in particular the normalized results describe the structure of the viral community in terms of relative abundance, and the read count output defines the richness and diversity of such community in the context of the metagenome of origin. The initial choice of the appropriate reference database is possible because the hierarchical databases built within ViromeScan contain sequences for DNA or DNA/RNA eukaryotic viruses, making the tool very adaptable to the needs of the user. Specifically, 92 genomes for the human DNA virus database, 664 for the human DNA/RNA virus database, 1646 for the eukaryotic DNA virus database and 4370 for the eukaryotic DNA/RNA virus database were employed in the construction of the tool. In addition, every ViromeScan user can create his/her own database for a customized analysis, including assembled sequences of unknown viruses, which could be useful to extend the taxon detection limit of the tool. Finally, another advantage of the ViromeScan tool is that the same metagenomic sample utilized to characterize bacteria, archeabacteria and eukarya within the microbiome, can be used for the viral profiling, opening new perspectives in metagenomic characterization studies.

**Fig. 2:**
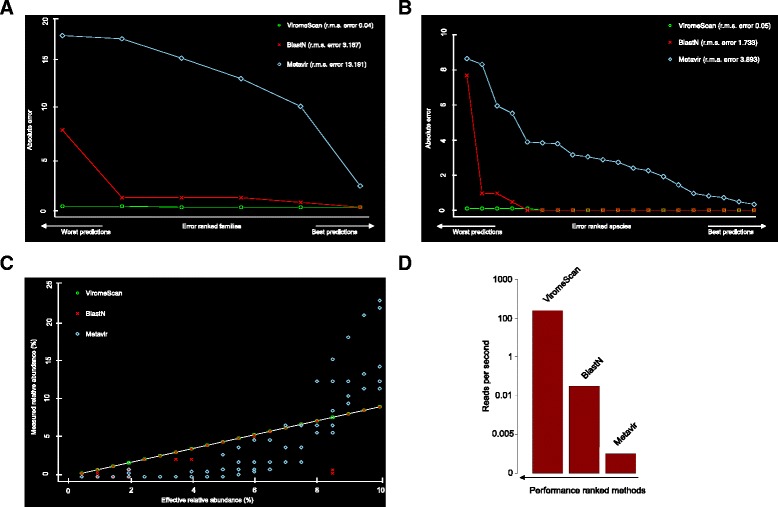
Comparison of ViromeScan to other existing methods. A total of five synthetic viral communities were used in order to compare ViromeScan with Metavir [22] and blastN [29]. Absolute and r.m.s. errors in assigning taxonomy at (**a**) family and (**b**) species level are shown. **c** Correlation between predicted and real relative abundance for the 5 non-evenly distributed mock communities. **d** Read rate for the tested tools on single CPU

**Fig. 3:**
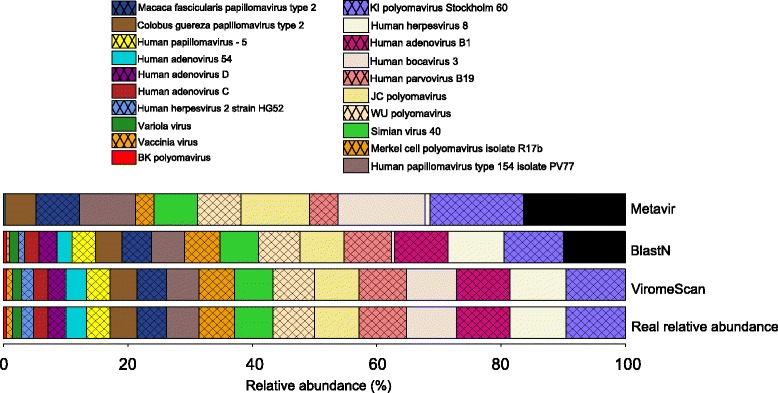
Comparison between the relative abundances of a single non-evenly distributed mock community as detected using Metavir [22], blastN [29] and ViromeScan, and its real composition. Black portions of the bars correspond to the unassigned viral fraction or erroneous viral assignment

We first evaluated ViromeScan performance in estimating the composition of viral communities using synthetic data. To this aim, we constructed 5 mock communities comprising reads from 20 different human DNA viruses, bacterial microorganisms and human genome, to simulate metagenomes including different domains as the intestinal microbiota. ViromeScan correctly mapped the majority of the reads and identified all the 20 viruses in the synthetic communities, accurately estimating their relative abundance at different taxonomic levels (r.m.s. errors 0.04 at family level and 0.05 at species level), with 100 % of the viral species within 1 % deviation from expected value and the best overall prediction (Pearson r > 0.999, species level Pearson *P* < 1 × 10^−22^), (Fig. 2a–c). ViromeScan was more accurate on all tested synthetic metagenomes than the other existing methods, with blastN showing the closest performance but substantially slower (Fig. 2). To be honest, several other tools for viral community characterization are available but they have been specifically designed to work with long sequences, or to detect open reading frames, which prevented their employment in our comparative analysis [19–21]. Furthermore, ViromeScan performed the classification at 140 reads per second on a standard single processor system, which was faster and more performing than other methods (Fig. 2d). The currently existing tools do not foresee filtering steps during the computational process, because they are designed to directly analyze viral reads. This fact constitutes a major limitation for the analysis of metagenomic samples, which usually contain a huge amount of bacterial and human reads. The strategy adopted by ViromeScan has been specifically studied to overcome this problem. In particular, two filtering steps, one for bacterial and one for human reads, have been introduced to reduce the dimensionality of the input dataset, saving time in the analysis computation. Additionally, yet importantly, ViromeScan showed a better mimicry of the abundance of the mock communities when compared to the other methods (Fig. 3). The better accuracy is probably due to the fact that bacterial and human reads are not filtered by the other approaches. By analyzing the assignment read by read, we deeply investigated how the non-filtering biases affected the performance of the other classification tools. Specifically, blastN failed to classify 50 % and 30 % of the reads belonging to *Human herpesvirus 2* strain HG52 and *Human adenovirus 54*, respectively. Furthermore, it assigned to a different strain the majority of the reads of *Human bocavirus 3* and *Vaccinia virus*. Analogously, Metavir failed to detect *Human herpesvirus 2* strain HG52, *Variola* and *Vaccinia virus*, *Human adenovirus C* and *D*. Moreover, it assigned to a different species the reads for *BK polyomavirus*, and overestimated the reads for *Parvoviridae* and *Polyomaviridae*. In these cases, the superior accuracy of ViromeScan is probably due to the unique “two-step” assignment process in the pipeline, which involves two consecutive alignments of the reads to the reference database. The first one is computed at the very beginning of the analysis to detect viral candidate reads. The second one is computed after the filtering processes, as validation and final assignment of the viral reads to the correct taxonomy. Notably, the “two-step” method is not used in the other existing tools [22, 29]. This uniqueness makes ViromeScan a very efficient tool in saving computational time, because it immediately skims the input reads, and at the same time permits a more accurate assignment of the viral sequences. Finally, by removing from the database the reference genomes closely related to those included in the mock communities, we evaluated the potential for viral discovery of the tool. According to our findings, ViromeScan was able to identify the correct genus of the *Human adenovirus* and *Human papillomavirus* species when their closest genome sequences were removed from the database, but it did not assign any human DNA virus when all the related genomes up to family level were deleted. For these reasons, ViromeScan can not be used as a classifier of viruses belonging to lineages that are completely missing in the database.

**Fig. 4:**
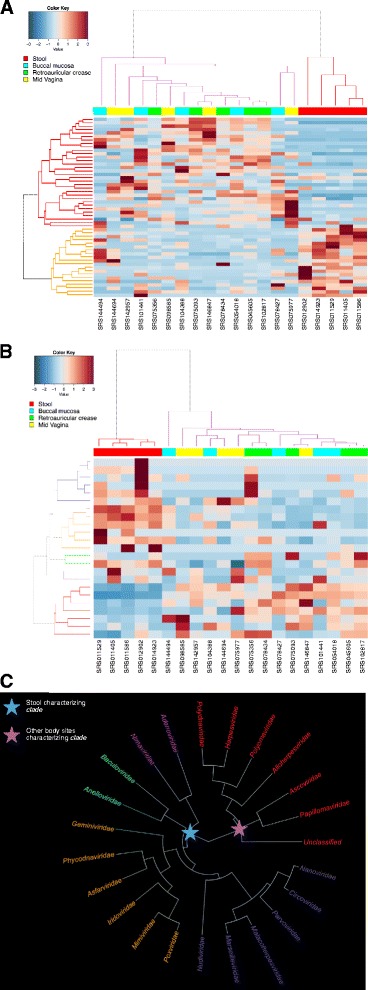
Different body sites reflect different virome configurations. Species (**a**) and families (**b**) level hierarchical Ward-linkage clustering based on the Spearman correlation coefficients of the viral profiles of 20 HMP samples [27] as determined by ViromeScan. Analysis was carried out considering all the families detected and species with at least 0.5 % of abundance in 25 % of samples. **c** Hierarchical Ward-linkage clustering of viral families generated characteristic clades, which discriminated the gut environment from the other body sites. The names of the families are colored according to the colors of the dendrogram (**b**)

**Fig. 5:**
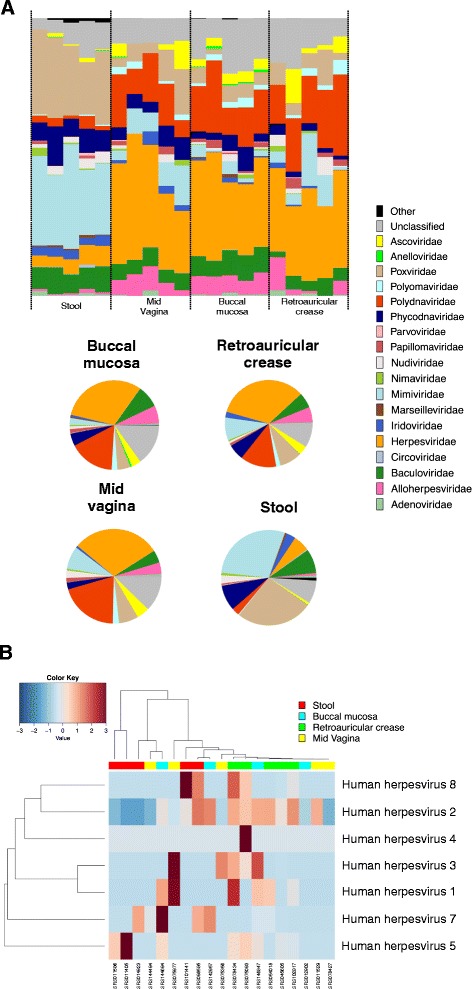
The eukaryotic virome at family level in an asymptomatic Western population, as predicted by ViromeScan. Analysis was carried out on 20 HMP samples [27] from 4 human body sites, including gut (stool), mouth (buccal mucosa), skin (retroauricular crease) and vagina (mid vagina). **a** The relative abundance of viral families for each HMP sample and the mean relative abundance for each body site are reported in the histograms and pie charts, respectively. **b** Hierarchical Ward-linkage clustering based on the Spearman correlation coefficients of 19/20 HMP samples containing members of the human *Herpesviridae* family

We next utilized ViromeScan to characterize the virome of metagenome samples from different body niches of people enrolled in the HMP [27], analyzing a total of 20 samples belonging to four human body sites: stool (representative of the gut ecosystem), mid vagina, buccal mucosa and retroauricular crease. ViromeScan detected 207 viral species from 22 viral families with abundance ≥ 0.5 % in at least one sample. The body site that showed the highest diversity was the retroauricular crease with 98 ± 10 (mean of viral species at ≥ 0.5 % ± sem), followed by gut (85 ± 3), buccal mucosa (48 ± 6), and vagina (42 ± 4). Looking at the genus-level diversity, we found a mean of 5.2 genera per sample, consistent with that detected in a previous study on 102 HMP samples (5.5 genera per sample) [35]. Thus, we investigated the hypothesis that different body sites reflect different virome profiles at family and species level through hierarchical clustering of the 20 samples (Fig. 4). Interestingly, the gut virome was consistently different from that of the other body sites (*P* < 0.05, Fisher’s exact test). In particular, it was characterized by *Geminiviridae*, *Phycodnaviridae*, *Asfarviridae*, *Iridoviridae*, *Mimiviridae*, *Adenoviridae*, *Nimaviridae*, *Baculoviridae*, *Anelloviridae*, *Nudiviridae*, *Marseilleviridae*, *Malacoherpesviridae*, *Parvoviridae*, *Circoviridae*, *Nanoviridae* and *Poxviridae* viral families. On the other hand, the other body sites shared some families, such as *Polydnaviridae*, *Herpesviridae*, *Polyomaviridae*, *Alloherpesviridae*, *Ascoviridae* and *Papillomaviridae* (P < 0.05, Fisher’s exact test). The differences are also displayed in terms of relative abundance in the histograms and pie charts of Fig. 5a. *Mimiviridae* and *Poxviridae* dominated the human gut eukaryotic virome, while *Herpesviridae* and *Polydnaviridae* were the most represented viral families in the other body sites. Notably, the relative abundances of HMP samples as determined by ViromeScan, were consistent with the results obtained by applying blastN (data not shown), and the viral taxa identified confirm the little available literature. In particular, *Papillomaviridae, Herpesviridae,* and *Polyomaviridae* have already been detected in the microbiota of vagina, skin and mouth [35], and *Adenoviridae*, *Anelloviridae* and *Circoviridae* in stool [9, 35]. Additionally, our findings on the gut samples led to the detection of *Megavirales* and other giant viruses that were not found in previous analyses of the human gut virome, probably due to the filtering approach used for virus isolation [9, 11], but have recently been isolated in human stool and other human samples through different approaches [15, 36–37]. Taken together, all these data confirm the applicability of ViromeScan to microbial communities and its suitability to metagenomic samples. By using ViromeScan read-based assignment against the in-house built human DNA virus database, we also provided the distribution of the *Herpesviridae* family across the different body sites with a species level resolution (Fig. 5b). Once more, these results highlighted the performance of ViromeScan, which could help bridge the general lack of information on the eukaryotic virome and its relationship with the host and the other microorganisms of the microbiome.

## Conclusions

ViromeScan provides the users with new perspectives in the virome characterization analysis. Shotgun metagenomics and RNAseq techniques are rapidly decreasing in cost and already supply a community-wide profiling of the bacterial, archeal and eukaryotic microbiome. By enabling an efficient detection of the viral counterpart from shotgun sequencing, ViromeScan extensively integrates the analysis of the microorganisms that inhabit the human body. Furthermore, ViromeScan can be applied to any environment as a tool for taxonomic profiling of the virome with resolution up to species level. An interesting and flexible aspect for users is that the pipeline of analysis can also be used with a customized database containing viral genomes of interest. However, this version of the tool remains blind to new viruses, which are not present in the database. For this reason, the pipeline will be integrated in the near future with an optional and parallel assembling step to identify unknown viruses within the metagenome. The ViromeScan databases of eukaryotic and human viruses will be periodically updated based on new genome releases.

## Availability and requirements

**Project name**: ViromeScan

**Project home page:**http://sourceforge.net/projects/viromescan/

**Operating systems**: Linux, OS X

**Programming language**: Bash, R, Perl, Java

**Other requirements**: Bowtie2, Bmtagger tools from NCBI, Picard tools

**Licence**: FreeBSD

**Any restriction to use by non-academics**: No

## Additional files

Additional file 1: IDs of the viral genomes included in the ViromeScan databases. (XLSX 115 kb) Additional file 2: List of the bacteria and corresponding genome IDs utilized in the bacterial read filtering procedure during the ViromeScan pipeline. (XLSX 11 kb) Additional file 3: List of the genome IDs and microorganisms used to construct the synthetic communities. (XLSX 37 kb) Additional file 4: List of the HMP samples [27] utilized to test the ViromeScan tool. (XLSX 43 kb)

## Abbreviations

BMTagger: human best match tagger
EAMMS: end anchored max scoring segments
GAAS: genome relative abundance and average size
HMP: human microbiome project
